# Parenting and Mental Illness: A Group for Mothers

**DOI:** 10.3389/fpsyt.2012.00071

**Published:** 2012-07-30

**Authors:** Yael Dvir

**Affiliations:** ^1^Child and Adolescent Psychiatry, Department of Psychiatry, University of Massachusetts Medical SchoolWorcester, MA, USA

## Parenting and Mental Illness

Popular conceptions of motherhood tend to exclude considerations of mental illness; nevertheless, women with mental illness do become pregnant and care for their children, a finding consistent across major diagnostic groups. Sixty-seven percent of women meeting criteria for severe and persistent mental illness over a 12-month period are mothers (Hinden et al., 2005). Most mothers living with mental illness and caring for their children describe motherhood as rewarding and central to their lives, but also report it as a difficult subject to discuss with their mental health providers (Diaz-Caneja and Johnson, 2004).

Families in which a mother has mental illness are families that may face multiple challenges. Mothers are at risk of psychiatric hospitalizations and child welfare involvement that may create separations and disruptions. Mothers living with mental illness are also more likely to be raising a child without a partner (Mowbray et al., 2001). Children with a mother with a mental illness have higher rates of psychiatric diagnosis and other psychosocial problems than children with mothers without mental illness (Hinden et al., 2005).

Existing services aimed at addressing parenting are primarily deficit-based, and most often available only in crisis (Hinden et al., 2005). Preventive interventions are rare and often withdrawn when the immediate crisis has resolved (Diaz-Caneja and Johnson, 2004).

## Family Options

Family Options (FO), an intervention developed to support parents living with mental illness and their families, is a partnership of University of Massachusetts Medical School Center for Mental Health Services Research, and Employment Options, Inc., a rehabilitation clubhouse model program in Marlborough, MA, USA. FO provides wraparound support to parents who have a history of mental health treatment and need support in their daily lives. FO provides prevention through care management for the entire family unit.

## Needs of Mothers with Mental Illness

Mothers with mental illness have many of the same challenges as all mothers including finding safe housing, obtaining transportation, securing employment, and managing finances. Issues that are specific to mothers with mental illness may include complying with treatment recommendations, particularly as it pertains to taking medications that have side effects, such as sedation, that might affect their ability to parent (Henry and Nicholson, 2005). As all parents, mothers with mental illness worry about their children's development. However, mothers with mental illness also worry about the impact of their mental illness on their children, and may view their children's difficult behavior, even if age appropriate, as a symptom of an emotional or behavioral disorder.

## A Support Group for Mothers

Although not a part of the original design of the FOs model, a support and education group for mothers participating in the program was initiated by this writer as a group therapy experience with a focus on support and education. Although the initial group design offered six hourly sessions with a clear content outline, it continued to meet weekly for 2 years and had up to six regular participants. A strength-based approach was introduced early on, and the mothers in the group were asked to think about their strengths as parents and share an example with the group. This was a difficult task for them; parents are often not aware of the strengths they have, but identifying and focusing on these womens’ strengths helped inspire confidence in their parenting.

The initial sessions focused on psychoeducation on normal child development and behavioral management, and actively involved the mothers in collaborative problem solving. Later sessions focused on topics such as food and meal preparation and enjoyable family activities. During the second year of the parenting group, as group members became more comfortable with each other, participants began to open up more and discussed their own illnesses, past hurtful relationships, and difficult decisions related to their children's needs. First person accounts were shared about the police coming into a home because a child is out of control, and of how difficult it is to decide to place a child in a residential treatment center or a foster home. Although the women's openness created a space for personal narratives, and an opportunity for members to listen to each other's struggles, it also shifted some of the focus from strengths and recovery to current and severe struggles. In order to maintain the strengths-based approach without removing a safe place to discuss intimate concerns, the group was introduced to a new concept. After discussing its rationale, group members and leaders agreed to begin each session by each answering the question: “what's better this week?” This ritual helped identify one's strengths amid severe difficulties.

Many mothers with mental illness become marginalized, leaving them feeling cut off and isolated (Oliver, 2004). We found that the group process may be one way to begin addressing this marginalization. Group leaders, as well as other members of the group were able to bear witness to woman's life experiences. Through this support and validation, the feeling of isolation begins to decrease.

This group experience emphasized that mothers with mental illness aspire to be the best parents they can be. As providers, it is our role to provide effective and preventative interventions that will enable mothers with mental illness to reach this goal. This approach benefits mothers living with mental illness and their children.
